# Visualization and Phospholipid Identification (VaLID): online integrated search engine capable of identifying and visualizing glycerophospholipids with given mass

**DOI:** 10.1093/bioinformatics/bts662

**Published:** 2012-11-18

**Authors:** Alexandre P. Blanchard, Graeme S. V. McDowell, Nico Valenzuela, Hongbin Xu, Sarah Gelbard, Martin Bertrand, Gary W. Slater, Daniel Figeys, Stephen Fai, Steffany A. L. Bennett

**Affiliations:** ^1^Ottawa Institute of Systems Biology, CIHR Training Program in Neurodegenerative Lipidomics, Biochemistry, Microbiology, and Immunology, University of Ottawa, Ontario K1H 8M5, ^2^Carleton Immersive Media Studio, Azrieli School of Architecture and Urbanism, Carleton University, Ontario K1S 5B6 and ^3^Physics, University of Ottawa, Ontario K1H 8M5, Canada

## Abstract

**Motivation:** Establishing phospholipid identities in large lipidomic datasets is a labour-intensive process. Where genomics and proteomics capitalize on sequence-based signatures, glycerophospholipids lack easily definable molecular fingerprints. Carbon chain length, degree of unsaturation, linkage, and polar head group identity must be calculated from mass to charge (m/z) ratios under defined mass spectrometry (MS) conditions. Given increasing MS sensitivity, many m/z values are not represented in existing prediction engines. To address this need, Visualization and Phospholipid Identification is a web-based application that returns all theoretically possible phospholipids for any m/z value and MS condition. Visualization algorithms produce multiple chemical structure files for each species. Curated lipids detected by the Canadian Institutes of Health Research Training Program in Neurodegenerative Lipidomics are provided as high-resolution structures.

**Availability:** VaLID is available through the Canadian Institutes of Health Research Training Program in Neurodegenerative Lipidomics resources web site at https://www.med.uottawa.ca/lipidomics/resources.html.

**Contacts:** lipawrd@uottawa.ca

**Supplementary Information:** Supplementary data are available at *Bioinformatics* online.

## 1 INTRODUCTION

The past 10 years have seen remarkable advances in high performance liquid chromatography, electrospray ionization mass spectrometry (MS). Coupled with careful biochemistry enabling the separation of membranes and, in some cases, membrane microdomains, adaptation of these technologies to the study of lipids is permitting comprehensive phospholipid profiling at the molecular level. The emerging field of lipidomics faces three main analytical challenges: (i) a paucity of bioinformatic tools for spectral analysis; (ii) the need for accurate lipid prediction algorithms before experimenters can proceed to empirical validation; (iii) a requirement for visual tools capable of displaying all theoretically possible lipid conformations in 2D and 3D. The most comprehensive tool kit has been developed by the LIPID MAPS Consortium representing >37 000 lipid species. The majority of their curated MS data has been generated using mouse leukemic monocyte-macrophage cells and, while extensive, does not yet represent all biological lipids. To our knowledge, none of the existing open-access web-based engines have capacity to predict identity of every m/z value detected in different MS spectra (Supplementary Table S1). Visualization and Phospholipid Identification (VaLID) is a comprehensive, simple to use, resource enabling rapid prediction of these tissue-specific lipid ‘unknowns’.

## 2 TOOL DESCRIPTION AND FUNCTIONALITY

### 2.1 Database content

The VaLID database contains exact and average masses (De Laeter *et al.*, 2003) for all theoretically possible: (i) phosphocholines; (ii) phosphoserines; (iii) phosphoethanolamines; (iv) glycerophosphates; (v) glyceropyrophosphates; (vi) glycerophosphoglycerols; (vii) glycerophosphoglycerolphosphates; and (viii) cytidine 5′-diphosphate 1,2-diacyl-*sn*-glycerols. The database includes *lyso*- and phospholipids with 1 to 30 carbons in each chain and up to six *cis* unsaturations. Calculations consider ester, alkyl ether and vinyl ether linkages. Numerical datasets are in Excel™ files.

### 2.2 Software requirements

VaLID requires a Java^TM^-enabled Internet browser.

### 2.3 Using VaLID to predict lipid identity

**Fig. 1.**
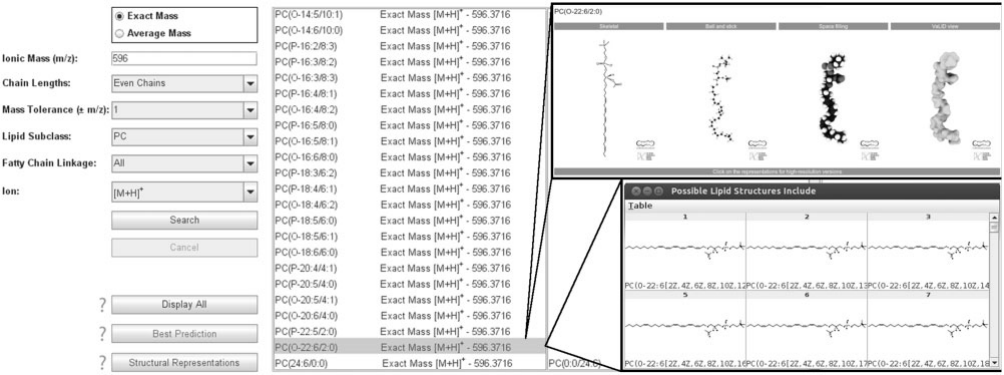
The VaLID interface

The VaLID search engine predicts lipid identity taking into consideration user-specific MS conditions (Fig. 1). The search engine is coded in Java™. The Excel files are read with JExcelApi. Users choose exact or average mass before inputting their m/z of interest. Simple pull-down menus restrict carbon chain lengths and linkages within phospholipid classes while considering appropriate ion type. Predictions appear in two panels: (i) *Possible Lipids Include* and (ii) *Possible Isomeric Lipids Include*. The first panel returns lipid identities with arbitrarily assigned *sn*-1 and *sn*-2 carbon chains. The second panel lists corresponding isomers. List position is ordered by lipid subclass (polar head group) and sorted by ascending (i) m/z; (ii) number of carbons (*sn*-1 chain); (iii) degree of unsaturation (*sn*-1 chain); (iv) number of carbons (*sn*-2 chain); and (v) degree of unsaturation (*sn*-2 chain). Nomenclature adheres to the LIPID MAPS classification system (Fahy *et al.*, 2011). To assist in decision-making, a ‘best guess’ feature is available whereby lipids in blue are considered ‘most likely’ based on the prevalence of constituent fatty acids in mammalian cells (Miyazaki and Ntambi, 2008). Lipids in red indicate that (i) the species is part of our Canadian Institutes of Health Research Training Program in Neurodegenerative Lipidomics (CTPNL)-curated database of neural lipids for which (ii) we have generated multiple high-resolution representations. Lipids in black represent theoretically possible combinations.

### 2.4 Displaying glycerophospholipid structures

To display all theoretically possible phospholipid conformations, users can highlight the lipid of interest and click on the ‘Display All’ button. The VaLID algorithm is confined to drawing cis double bonds separated by a minimum of two carbons. Using JExcelApi, VaLID calculates where each atom should be in 2D-space and generates 2D representations displayed using ChemAxon’s Marvin View 5.5.1.0. Marvin View enables the user to toggle through structural views and save each species in a variety of formats. Chemical structures are drawn in accordance to the standards developed by the LIPID MAPS consortium (Fahy *et al.*, 2011). The ‘Best Prediction’ button displays only those lipid species found in VaLID’s *Predicted to be Common* database. The ‘Structural Representations’ button displays lipids that are part of VaLID’s curated *Structural Representations* database identified in neural tissue by CTPNL researchers. These species can be viewed in high-resolution as (i) 2D skeletal models; (ii) 3D ball and stick models; (iii) space filling models; or (iv) rendered ‘VaLID view’ models. VaLID view models were assembled in ChemDraw3D®, translated into a 3D model where each atom was marked by an *x*, *y* and *z* coordinate and exported into Autodesk® Maya® v2012. Rigid and dynamic models were derived using Maya® nParticles, converted into smooth polygonal meshes. These meshes were directed to the original *x*, *y* and *z* coordinates and imported as points in space to recapitulate the original molecular structure in an abstracted, organic, form. Resulting VaLID view models are available for download as rigid polygons. They are also available on request fitted with a rig of movable joints between atoms, a process typically used by graphic artists to animate human or animal characters, to facilitate membrane reconstruction and modelling.

## 3 CONCLUSION

VaLID is a web-based application linking a convenient search engine, a phospholipid database and multiple visualization features for identification and dissemination of large-scale lipidomic datasets. VaLID returns all theoretically possible species based on m/z and user-defined MS conditions. The user is cautioned that VaLID includes lipids (and isomeric bond configurations) that may not be biologically relevant. Investigators are encouraged to mine these lists for species most relevant to their specific biological system for subsequent validation. To assist in decision-making, a ‘best guess’ feature is available to focus on lipids predicted to be common based on the prevalence of the fatty acid chains in mammalian cells. Every theoretical conformation (in *cis* configuration) for each species can be viewed in 2D and 3D. Curated species can also be downloaded in multiple high-resolution representations for further visualization and model production.

*Funding*: This resource was funded by CIHR-MOP 89999/CIHR/Institute of Aging-TGF 96121 (to D.F., S.F. S.B.); NSERC CREATE (to D.F., G.S.); Autodesk Research, CFI (to S.F.). A.B., G.M., N.V., S.G., M.B. and H.X. received CTPNL, NSERC CREATE, FRSQ and MITACs awards.

*Conflict of Interest*: none declared.

## Supplementary Material

Supplementary Data
