# Supplementary material for: Visualization and Phospholipid Identification (VaLID): online integrated search engine capable of identifying and visualizing glycerophospholipids with given mass
Source: Bioinformatics. 2012 Nov 18;29(2):284–5. doi: 10.1093/bioinformatics/bts662 (PMC3546797; doi:10.1093/bioinformatics/bts662)
Supplement: Supplementary Data [file supp_29_2_284__index.html]

Visualization and Phospholipid Identification (VaLID): online integrated search engine capable of identifying and visualizing glycerophospholipids with given mass — Supplementary Data 

# Visualization and Phospholipid Identification (VaLID): online integrated search engine capable of identifying and visualizing glycerophospholipids with given mass

## Supplementary Data

files

**Files in this Data Supplement:**

- Supplementary Data - pdf file
- Supplementary Data - docx file
