# Supplementary material for: Visualization and Phospholipid Identification (VaLID): online integrated search engine capable of identifying and visualizing glycerophospholipids with given mass
Source: Bioinformatics. 2012 Nov 18;29(2):284–5. doi: 10.1093/bioinformatics/bts662 (PMC3546797; doi:10.1093/bioinformatics/bts662)
Supplement: Supplementary Data [file supp_bts662_VaLIDuserguide.pdf]

VaLID: Visualization and Phospholipid Identification

a glycerophospholipid m/z prediction database

User guide
version 1.0.1

Developed by:

Graeme S.V. McDowell, Alexandre P. Blanchard, and Nico Valenzuela as part of the Canadian Institute of Health Research (CIHR) Training Program in Neurodegenerative Lipidomics.

Supported by:

A Strategic Training Initiative in Health Research grant from CIHR and the Institute of Aging (TGF-96121) to Stephen Fai, Daniel Figeys, and Steffany A.L. Bennett

Online at:

https://www.med.uottawa.ca/lipidomics/resources.html

For tech and resource support:

Please email: lipawrd@uottawa.ca

CIHR IRSC uOttawa Sunnybrook HEALTH SCIENCES CENTRE Carleton UNIVERSITY UNIVERSITY OF TORONTO CIHR Training Program in Neurodegenerative Lipidomics

I. What is VaLID? 3
II. Using VaLID 4
1. VaLID requirements 4
2. Running VaLID 5
3. Searching VaLID's glycerophospholipid database 6
4. Displaying glycerophospholipid structures 7
5. Downloading glycerophospholipid structures 9
6. Exiting the application 12
7. Supplementary information 12
III. Citing VaLID in a scientific paper 13
1. How do I cite use of VaLID in publications? 13
2. Citation information: 13
IV. References 13

I. What is VaLID?

"Visualization and Phospholipid Identification" (VaLID) is a web-based application linking a convenient search engine, a phospholipid database, and multiple visualization features for identification and dissemination of large-scale lipidomic datasets. Given the rapidly evolving nature of the lipidomic field, many mass to charge (m/z) values are not yet represented in other open-access web-based engines. To address this need, VaLID returns all theoretically possible species based on m/z and user-defined MS conditions in addition to highlighting species identified by members of the Canadian Institutes of Health Research (CIHR) Training Program in Neurodegenerative Lipidomics (CTPNL). The intent is to complement existing curated databases by enabling users to predict the identity of 'new' species detected in their particular mass spectrometry (MS) datasets and thus proceed more rapidly to validation. While useful for lipid discovery, the user is cautioned that VaLID includes lipids (and isomeric bond configurations) that may not be biologically relevant. Investigators are encouraged to mine these lists for species most relevant to their specific biological system for subsequent validation. To assist in decision-making, a "best guess" feature is available whereby lipids listed in blue are predicted to be the most likely based on the prevalence of constituent fatty acid chains in mammalian cells (Miyazaki and Ntambi, 2008). Every theoretical conformation (in *cis* configuration) for each species can be viewed in 2D and 3D. In this version, curated species detected in brain tissue by members of the Canadian Institutes of Health Research (CIHR) Training Program in Neurodegenerative Lipidomics (CTPNL) can also be downloaded in multiple high-resolution representations for further visualization and model production.

In version 1.0.1, the VaLID search engine encompasses:

PC: glycerophosphocholines
PS: glycerophosphoserines
PE: glycerophosphoethanolamines
PA: glycerophosphates
PG: glycerophosphoglycerols
PGP: glycerolphosphoglycerolphosphates
PPA: glyceropyrophosphates
CDP-DG: cytidine 5'-diphosphate 1,2-diacyl-*sn*-glycerols

VaLID follows the LIPID MAPS classification and nomenclature system in lipid naming (Fahy et al., 2011).

II. Using VaLID

1. VaLID requirements

1.1. An Internet connection is required.

1.2. VaLID is accessible at https://www.med.uOttawa.ca/lipidomics/resources.html

1.3. VaLID has been tested to work with Java^{TM} v7 on the following web browsers:

- 1.3.1. Safari: v6.0 - OS X
- 1.3.2. Opera: v12.02 - Windows; v12.0.2 - OS X
- 1.3.3. Firefox: v14.0.1 (not compatible with v15.0.1) - Windows; v15.0.1 - OS X
- 1.3.4. Internet Explorer: v9.0.8112.16421 - Windows
- 1.3.5. Chrome: 21.0.1180.89 - Windows v21.0.1180.89 - OS X

1.4. The help feature and structure drawing features of VaLID require that the user enable pop-up windows for the site.

1.5. Before starting VaLID, verify that Java^{TM} is installed on your computer. Java is a programming language required for VaLID to function and it is free to download. Go to http://java.com/en/download/installed.jsp and click on “Verify Java version.”

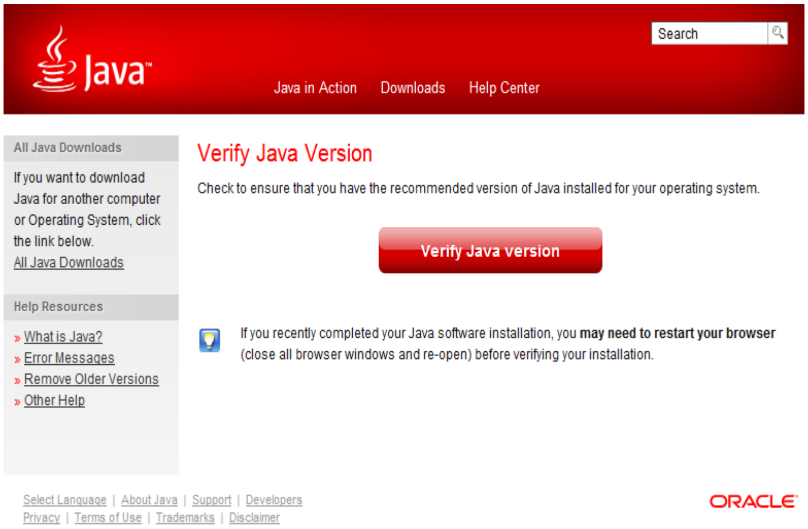

1.6. If you do not have Java^{TM} installed, follow the instructions on the Java website to install one. Alternatively,

For Microsoft Windows, the latest Java version can be found at: http://java.com/en/download/manual.jsp.

For Apple OS X, Java can be updated through Software Update (for more information concerning Software Update, go to: http://support.apple.com/kb/HT1338?viewlocale=en_US/).

2. Running VaLID

2.1. You will be asked to accept the security certificate for our lipid database. This message will not appear again once you have accepted the certificate.

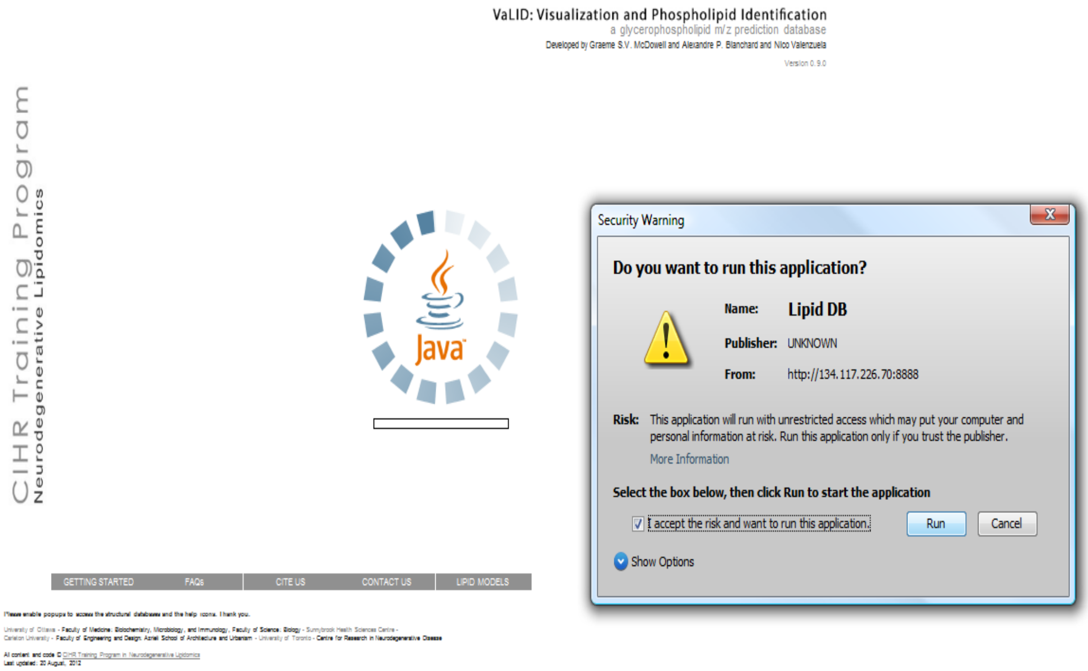

In some browsers, VaLID will not start until you click once in the centre of the browser window to initiate Java. Once properly loaded, this window will appear:

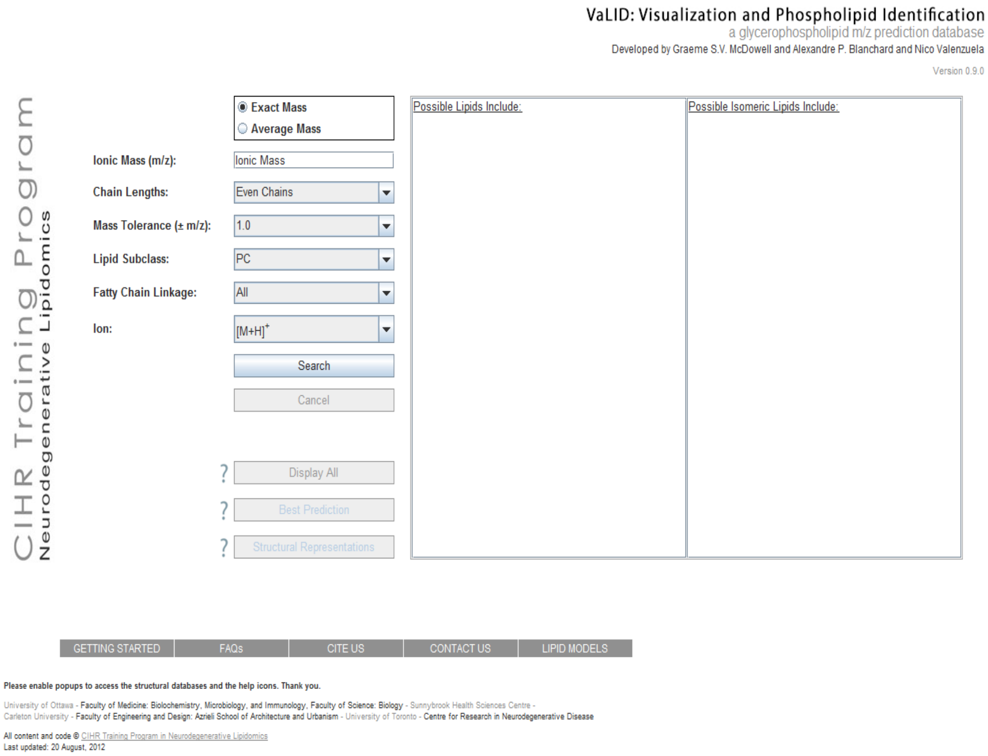

3. **Searching VaLID's glycerophospholipid database**
   3.1. Choose Exact Mass or Average Mass
   3.2. Input the m/z of your lipid of interest into the "Ionic Mass" field.
   3.3. Use the drop-down menus to select:
      3.3.1. Chain Lengths: Even Chain, Odd Chain or All
      3.3.2. Mass Tolerance: ± 0.0001 to ± 2
      3.3.3. Lipid Subclass: PC, PS, PE, PA, PPA, PG, PGP, or CDP-DG.
      3.3.4. Fatty Chain Linkage: All, Alkenyl-Acyl, Alkyl-Acyl, Diacyl, Dialkyl, or Mono-Acyl, Alkyl and Alkenyl.
      3.3.5. Lipid ions: $[M+H]^+$, $[M+K]^+$, $[M+Li]^+$, $[M+Na]^+$, $[M-H]^-$ or [M(Neutral)].
      Note: The glycerophospholipid database contains phospholipids with fatty chains from 0 to 30 carbons and up to 6 cis unsaturations. The differential combinations of these individual fatty acyl chains at the *sn*-1 and *sn*-2 positions with ester, alkyl ether, or vinyl ether linkages lead to the formation of the majority of biologically relevant lipids.
   3.4. When all of the search options are selected based on your MS conditions, click on the "Search" button or press the "Enter" key on the keyboard.
      Note: The program currently does not support parallel searches of multiple lipid species in different tabs of the same web browser.
   3.5. Initial searches may take longer than a minute, depending upon the connection speed, and search options selected. Why? When a new search is started the program loads all the lipids with the mass you are searching for into memory, as well as lipids with mass ratios 25 m/z above and below the target mass. This cached buffer of lipids with 50 m/z of your target range allows for subsequent searches close to the original search to occur much faster than if the program reloaded the database each time.
   3.6. The possible lipids based on the search criteria will appear in two panels, (i) "Possible Lipids Include" and (ii) "Possible Isomeric Lipids Include."

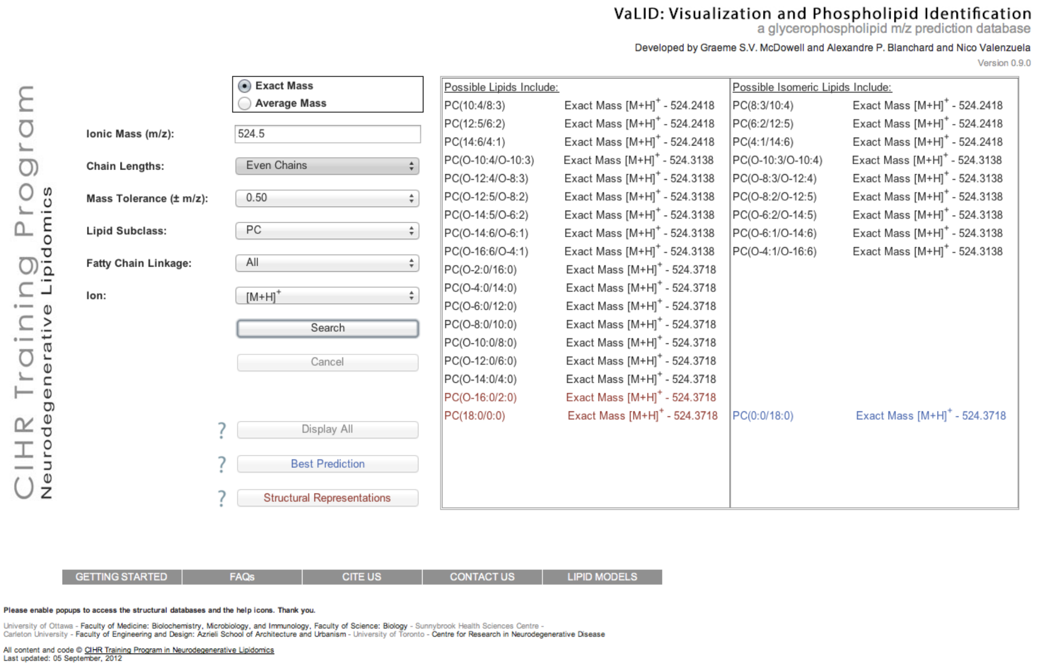

3.7. The left panel (Possible Lipids Include) shows lipid predictions with different chains at *sn*-1 and *sn*-2 positions of the glycerol backbone while the right panel (Possible Isomeric Lipids Include) shows corresponding isomers with the same chains but at different positions on the glycerol backbone. Lipid location in these lists is not sorted by biological significance but grouped according to lipid subclass (polar head group) and ascending (i) m/z, (ii) number of carbons (*sn*-1 chain), (iii) degree of unsaturation (*sn*-1 chain), (iv) number of carbons (*sn*-2 chain), and (v) degree of unsaturation (*sn*-2 chain).

3.8. 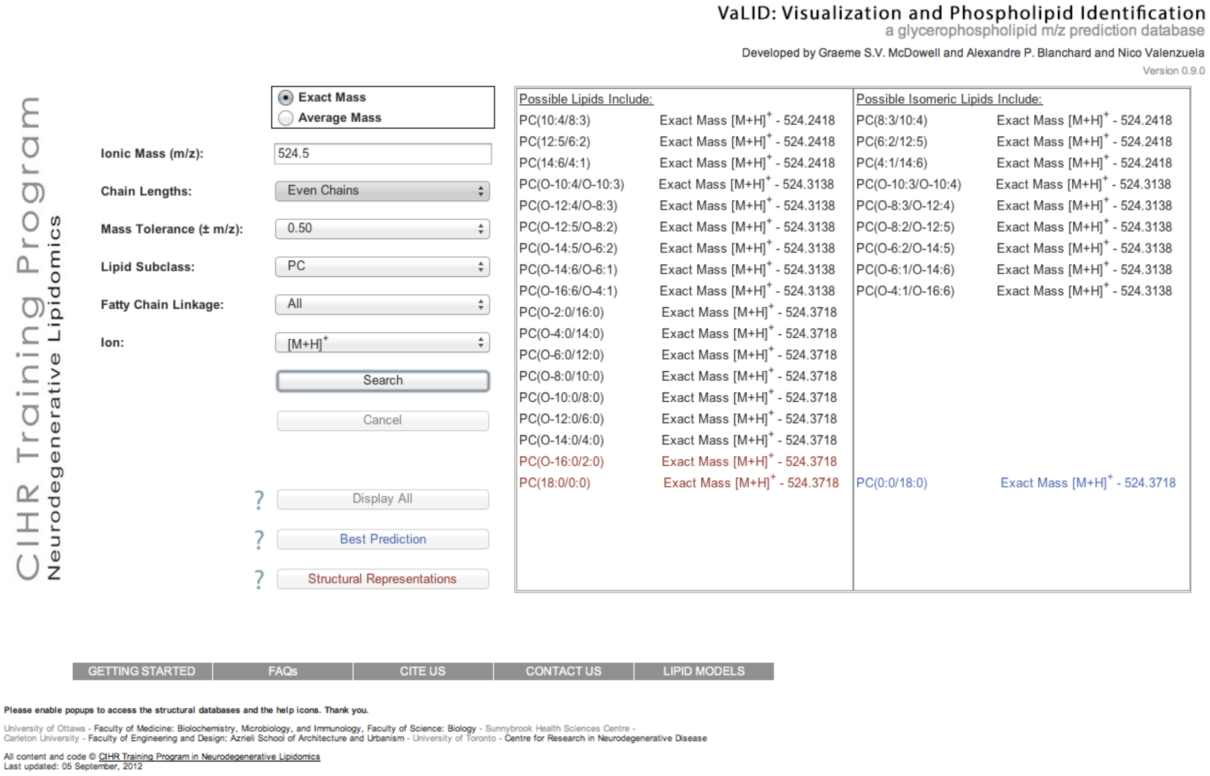

3.9. Each lipid entry is selectable (click once to highlight a given species). Name and m/z value can be copied by pressing "CTRL + C" and pasted by "CTRL + V".

4. Displaying glycerophospholipid structures

4.1. All possible lipid structures can be viewed in a new pop-up window. Make sure that pop-up windows are allowed on your web browser. The text colour helps the user to restrict the number of structures presented:

4.1.1. Lipids in black represent theoretically possible combinations.

4.1.2. Lipids in blue are predicted to be the most likely based on prevalence of fatty acid chains in mammalian cells (Miyazaki and Ntambi, 2008).

4.1.3. Lipids in red indicate that (i) the lipid is part of our CTPNL curated database of lipids detected by MS methodolgies in brain tissue or neural cells (neurons, astrocytes, or oligodendrocytes including precursor populations) and (ii) we have generated multiple high-resolution representations for further visualization and model production.

4.2. To view all possible lipid structures, click once on the lipid of interest. Then click:

4.2.1. The “Display All” button to display all theoretically possible lipid species in 2D. All entries in the database can be visualized in this mode.
Note: Displayed species contain only cis double bonds separated by a minimum of 2 carbons.

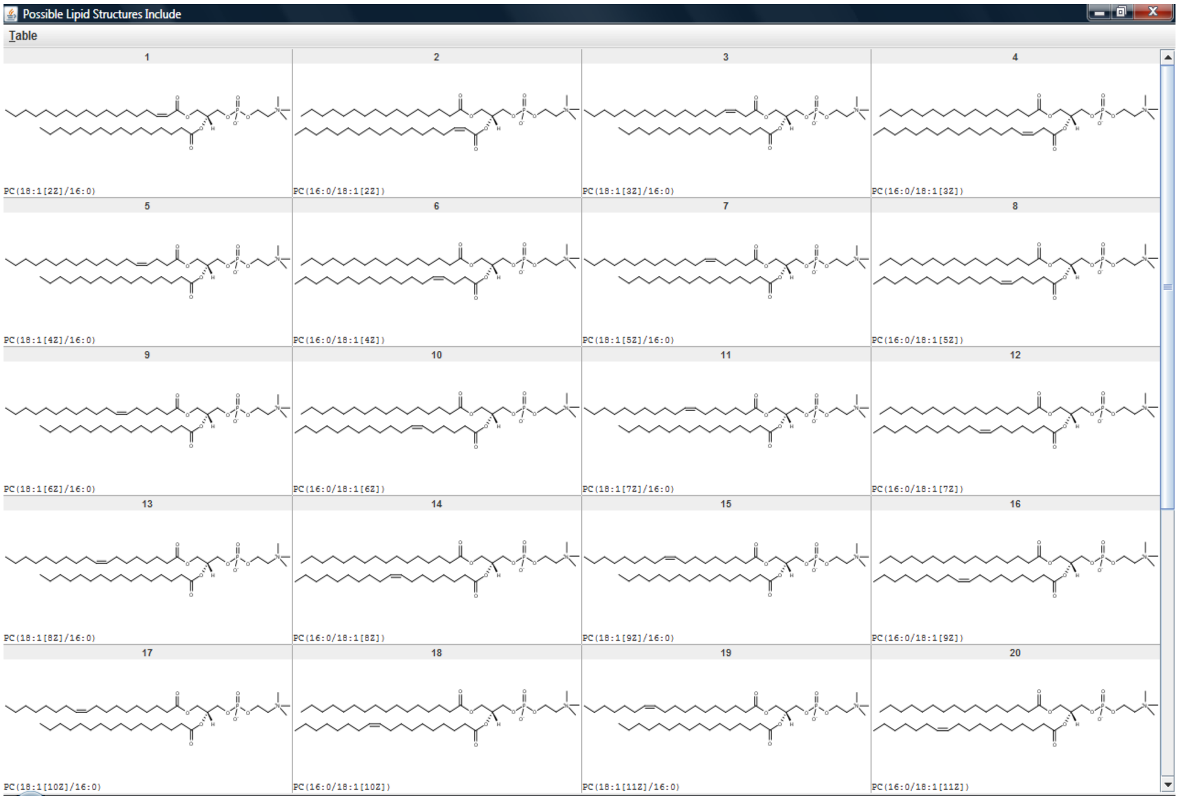

4.2.2. The “Best Prediction” button displays only lipid species that are predicted to be the best guess. This function works only with lipid entries in blue font.
Note: Lipids in VaLID’s “Predicted to be Common” database were identified based on the relative abundance of certain fatty acid chains in mammalian cells (Miyazaki and Ntambi, 2008). If both of the fatty acid chains at the *sn-1* and *sn-2* positions or if a single chain in a lysophospholipid are considered common, the lipid is included in the Predicted Common database and appears in blue.

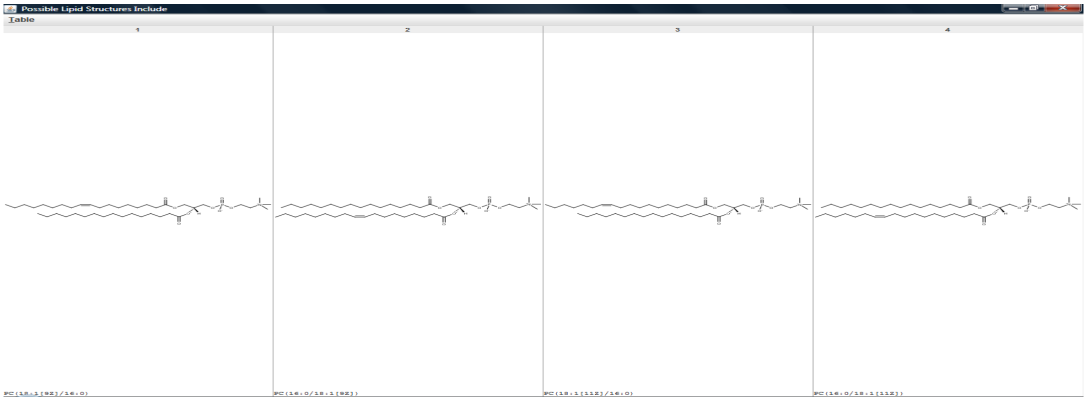

4.2.3. The “Structural representations” button displays lipids that are part of our CTPNL curated database of neural lipids. This button displays a known structural representation present in mammalian membranes as a (i) 2D skeletal model, (ii) 3D “Ball and stick” model, (iii) “Space filling” model and (iv) our own rendered “VaLID view” model.

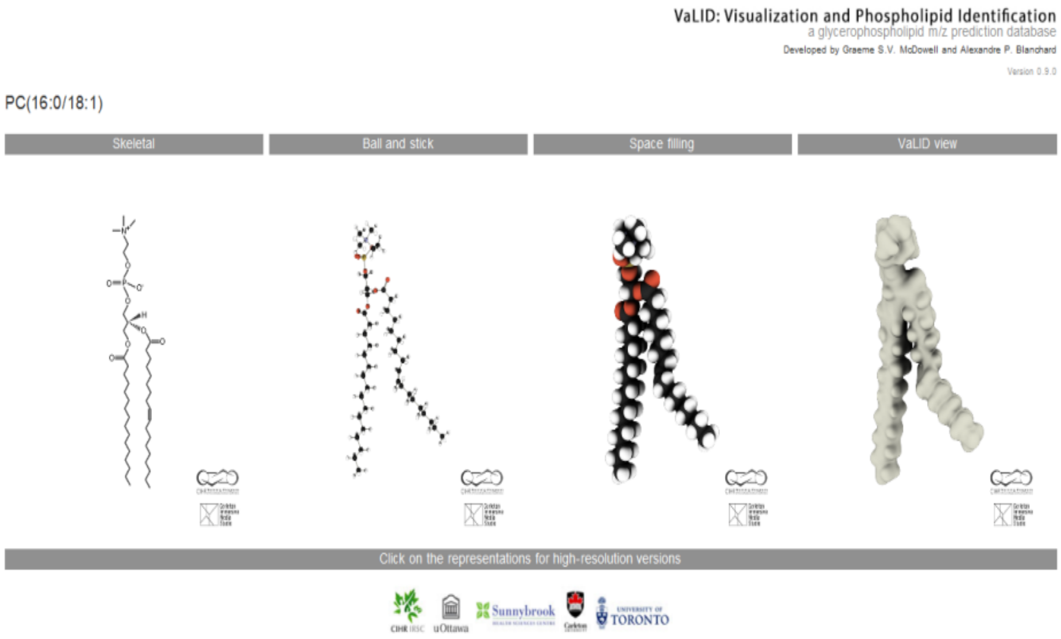

5. Downloading glycerophospholipid structures

5.1. To save a lipid structure from the “Display All” or “Best Prediction” windows, double-click on a structure of interest in the pop-up window displaying all possible configurations. A new window will open with the selected lipid structure.

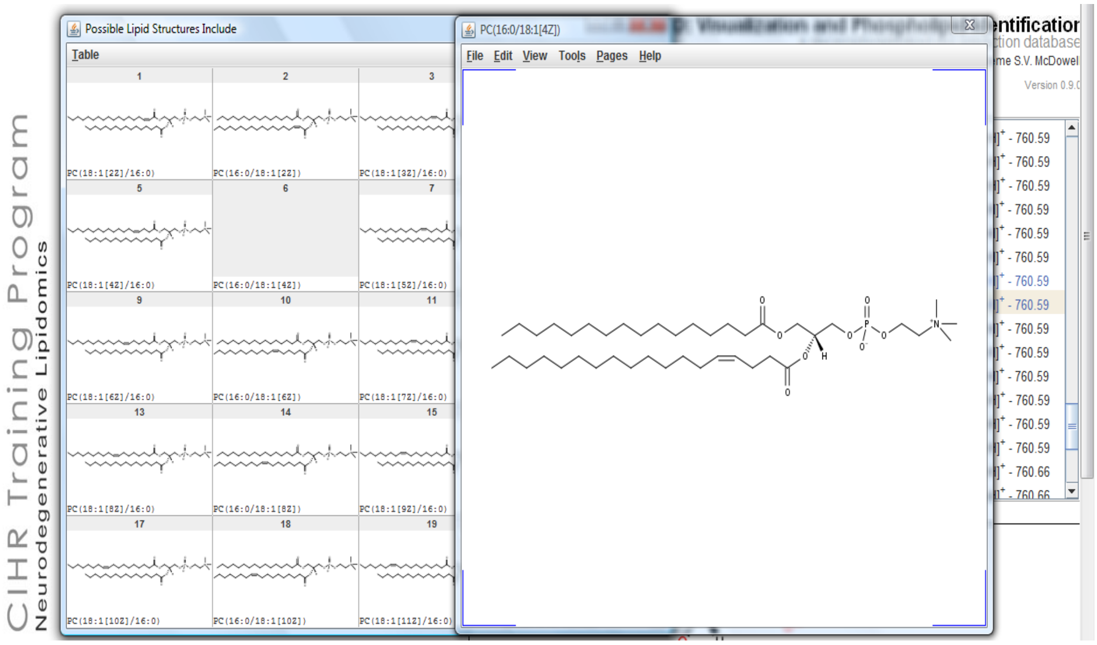

5.1.1. The image can now be saved in low resolution in two different file types:

5.1.1.1. As an image file, go to the “File” menu, select “Save As Image.” You will have a choice of the following formats: joint photographic experts group (.jpg, .jpeg), portable document format (.pdf), portable network graphics (.png), portable pixmap (.ppm), scalable vector graphics (.svg, .svgz), Windows BMP (.bmp) and Windows enhanced metafiles (.emf).

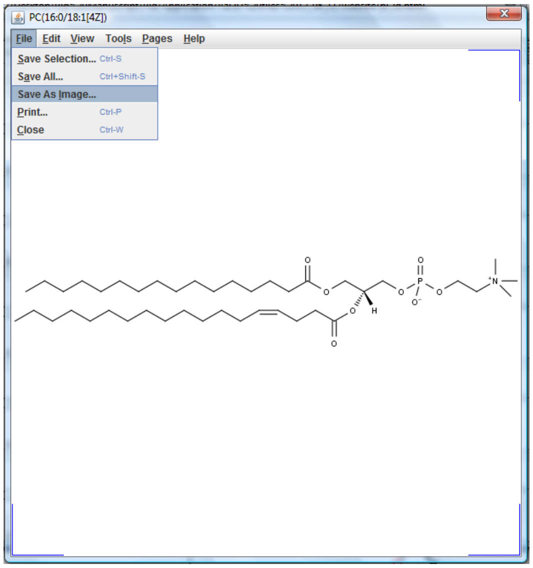

5.1.1.2. As a chemical structure information file that is compatible with ChemDraw and Marvin, go to the “File” menu and choose “Save Selection.” A save dialogue box will appear, allowing you to save the file on your hard drive. You can save the file as one of many file formats, including ChemAxon Marvin (.mrv), MDL Mol file (.mol) and CDX file (.cdx, suitable for ChemDraw) formats.

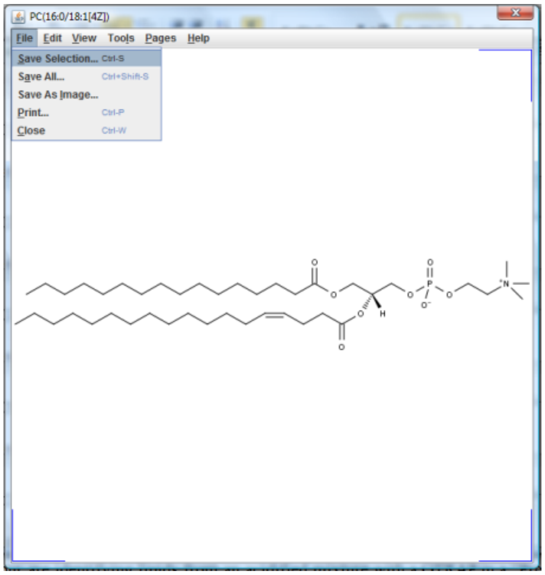

5.1.2. To save the high quality (300 dpi) lipid structures present in our "Structural Representation" databases, click on the model of interest: (i) 2D skeletal model, (ii) 3D "Ball and stick" model, (iii) "Space filling" model, or (iv) our own rendered "VaLID view" model.

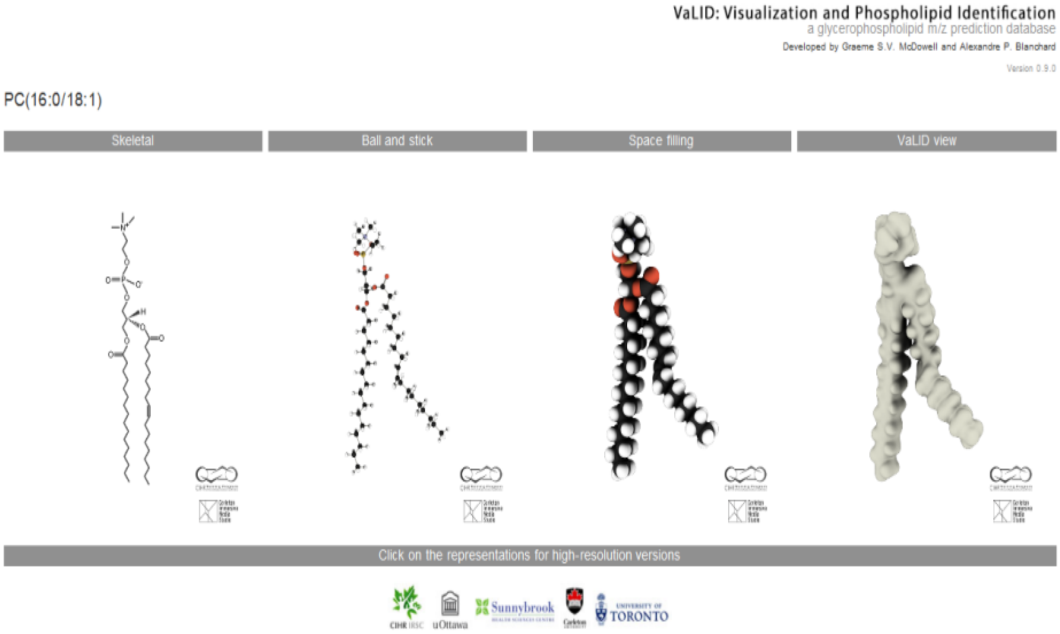

5.2. A new window will open in the web browser with the name of the species you have selected. Right click (or double click) on the image and click on "Save Image" or choose "Save As" from the browser menu.

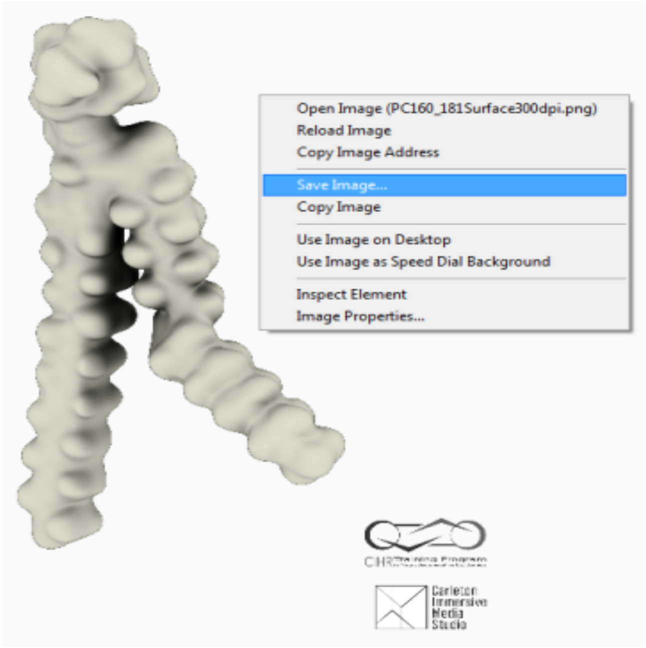

6. **Exiting the application**
    6.1. To close the application, navigate away from the page.
    6.2. To free up all resources the application has used, quit/close your browser. By closing your browser, you close Java and any system resources used by the application.

7. **Supplementary information**
    7.1. Coding:
        7.1.1. The numerical database is contained in Excel™ files.
        7.1.2. The search engine and applet are coded in Java™.
        7.1.3. The Excel files are read with JExcelApi.
        7.1.4. The 2D structures are visualized with ChemAxon's Marvin View plugin.
        7.1.5. The structural representations have been created with ChemDraw® and Autodesk® Maya® of curated lipid species identified by our CTPNL researchers by MS methodologies in neural tissue. The models have been developed by CTPNL researchers for purposes of representation and dissemination of simulation readouts. Of these models, VaLID view representations can be used as components of comprehensive membrane structures. Each lipid is first assembled in ChemDraw 3D. Structures drawn in 2D with ChemDraw's drawing tools are automatically translated into a 3D model in an adjacent panel where each atom of the visualized molecule are marked by an x, y and z coordinate. These points are then exported into the 3D modeling and animation software package, Autodesk Maya v2012. Rigid and dynamic VaLID view models are derived using Maya's nParticles tools. Typically, these tools are used in the animation of dynamic systems such as fire, smoke and water. nParticles are easily converted into smooth and organic polygonal meshes. By directing these particles to the x, y, and z coordinates originally generated in ChemDraw 3D and imported in Maya as points in space, a mesh that recapitulates the original molecular structures in an abstracted, organic, form. Here, VaLID view models are available for download as rigid polygon models however in Maya, we have fitted each species between each atom with a rig of movable joints, a process typically used by graphic artists to animate human or animal characters. By creating a skeleton embedded within the mesh model, we have enabled each of these structures to move in a 'biological' or dynamic way. Joint attributes are modified to restrict or limit movement (i.e., in the case of a double bond) and can be reassembled into membrane form.

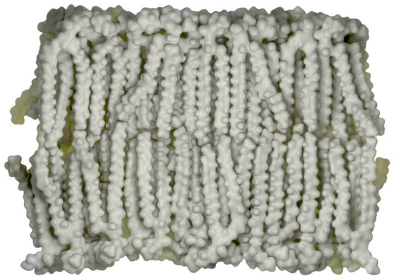

III. Citing VaLID in a scientific paper

1. **How do I cite use of VaLID in publications?**
   1.1. When citing predictions performed using the program, use this example as a guide: Glycerophospholipid identities were predicted based on m/z and appropriate ion type taking into consideration the mass tolerance of the QTRAP 5500 [replace with name of appropriate mass spectrometer] using VaLID version 1.0.1 (Blanchard et al., 2012).
   1.2. When citing use of structural images, use this example as a guide: "Structural images were obtained from the VaLID structural database version 1.0.1 (Blanchard et al., 2012)."

2. **Citation information:**
   Blanchard AP, McDowell GSV, Valenzuela N, Xu H, Gelbard S, Bertrand M, Slater GW, Figeys D, Fai S, Bennett SAL (2012) Visualization and Phospholipid Identification (VaLID): An online integrated search engine capable of identifying and visualizing glycerophospholipids with given mass. Bioinformatics (in press)
   http://134.117.226.70:8888/valid_index.html

IV. References

Fahy E, Cotter D, Sud M, Subramaniam S (2011) Lipid classification, structures and tools. Biochim Biophys Acta 1811:637-647.

Miyazaki M, Ntambi JM (2008) Fatty acid desaturation and chain elongation in mammals. In: Biochemistry of Lipids, Lipoproteins and Membranes (Vance DE, Vance JE, eds), pp 191-211. Oxford: Elsevier.
