# Supplementary material for: Visualization and Phospholipid Identification (VaLID): online integrated search engine capable of identifying and visualizing glycerophospholipids with given mass
Source: Bioinformatics. 2012 Nov 18;29(2):284–5. doi: 10.1093/bioinformatics/bts662 (PMC3546797; doi:10.1093/bioinformatics/bts662)
Supplement: Supplementary Data [file supp_bts662_SupplementaryTable1.docx]

**Table 1: m/z and spectral databases and prediction engines for lipid identification**

| **Tool** | **Availability** | **References** |
| --- | --- | --- |
| lipID | email to: [mass-spec@fz-borstel.de](mailto:mass-spec@fz-borstel.de) | (Hubner, et al., 2009) |
| LIPID MAPS Structure Database (LMSD) | <http://www.lipidmaps.org/data/structure/> | (Fahy, et al., 2011) |
| Lipid Mass Spectrum Analysis (LIMSA) | <http://www.helsinki.fi/science/lipids/software.html> | (Haimi, et al., 2009) |
| Lipid Qualitative/Quantitative Analysis (LipidQA) | <http://msr.dom.wustl.edu/research/Downloadable_Software.htm> | (Song, et al., 2007) |
| LipidBank | <http://lipidbank.jp/> | (Watanabe, et al., 2000) |
| LipidomeDB Data Calculation Environment  (DCE) | <http://lipidome.bcf.ku.edu:9000/Lipidomics> | (Zhou, et al., 2011) |
| LipidXplorer | <https://wiki.mpi-cbg.de/wiki/lipidx/index.php/Main_Page> | (Herzog, et al., 2012) |
| multiWayCCA | <http://research.ics.aalto.fi/mi/software/multiWayCCA/> | (Huopaniemi, et al., 2010) |

**References**

Fahy, E.*, et al.* (2011) Lipid classification, structures and tools, *Biochim Biophys Acta*, **1811**, 637-647.

Haimi, P.*, et al.* (2009) Instrument-independent software tools for the analysis of MS-MS and LC-MS lipidomics data, *Methods Mol Biol*, **580**, 285-294.

Herzog, R.*, et al.* (2012) LipidXplorer: a software for consensual cross-platform lipidomics, *PLoS One*, **7**, e29851.

Hubner, G., Crone, C. and Lindner, B. (2009) lipID--a software tool for automated assignment of lipids in mass spectra, *Journal of mass spectrometry : JMS*, **44**, 1676-1683.

Huopaniemi, I.*, et al.* (2010) Multivariate multi-way analysis of multi-source data, *Bioinformatics*, **26**, i391-398.

Song, H.*, et al.* (2007) Algorithm for processing raw mass spectrometric data to identify and quantitate complex lipid molecular species in mixtures by data-dependent scanning and fragment ion database searching, *Journal of the American Society for Mass Spectrometry*, **18**, 1848-1858.

Watanabe, K., Yasugi, E. and Oshima, M. (2000) How to search the glycolipid data in LIPIDBANK for Web: the newly developed lipid database, *Japan Trend Glycosci Glycotechnol*, **12**, 175-184.

Zhou, Z.*, et al.* (2011) LipidomeDB data calculation environment: online processing of direct-infusion mass spectral data for lipid profiles, *Lipids*, **46**, 879-884.

**Acknowledgements**

We gratefully acknowledge the critical comments of Dr Alex Brown (uVanderbilt), Dr Theodore Perkins (uOttawa), Dr Leigh-Anne Swayne (uVictoria), Dr Shawn Whitehead (uWestern), Dr Jeff Smith (Carleton), Matthew Cooke (uOttawa), and Marc Léonard (CIMS) as well those of our beta-testers David Myers (uVanderbilt), Dattatreya Mellacheruvu (uMichigan), Avinash Shanmugam (uMichigan), Laura Hamilton (McGill), Matthew Granger (uOttawa), Graham Mazereeuw (Sunnybrook), and Deborah Swartz (uToronto).
